# Early stage neuroglobin level as a predictor of delayed cerebral ischemia in patients with aneurysmal subarachnoid hemorrhage

**DOI:** 10.1002/brb3.1547

**Published:** 2020-02-05

**Authors:** Chenyu Ding, Dezhi Kang, Pengqiang Chen, Ziliang Wang, Yuanxiang Lin, Dengliang Wang, Zhangya Lin, Jianjun Gu

**Affiliations:** ^1^ Department of Neurosurgery The First Affiliated Hospital of Fujian Medical University Fuzhou China; ^2^ Department of Neurosurgery Zhengzhou University People's Hospital Henan Provincial People's Hospital Zhengzhou China

**Keywords:** aneurysmal subarachnoid hemorrhage, delayed cerebral ischemia, neuroglobin, predictor, stroke, World Federation of Neurosurgical Societies Grade

## Abstract

**Background:**

The neuroglobin (Ngb) is well recognized as a potential biomarker for the hypoxic‐ischemic brain injury. However, connection between Ngb and delayed cerebral ischemia (DCI) following aneurysmal subarachnoid hemorrhage (aSAH) is still unclear.

**Objective:**

To investigate the relationship between early stage Ngb level of aSAH patient and the occurrence of DCI.

**Methods:**

We evaluated 126 aSAH patients who were enrolled into a prospective observational cohort study. Serum Ngb level on days 1, 2, 3, 5, and 7 after aSAH were determined using a commercial enzyme‐linked immunosorbent assay kit. The relationship between Ngb level and DCI was analyzed.

**Results:**

Forty‐six (36.5%) aSAH patients experienced DCI. Patients with DCI had significantly higher Ngb levels than those without (*p* < .001). Multivariate model analysis revealed that day 3 Ngb level remained a significant factor after adjusting for World Federation of Neurosurgical Societies (WFNS) grade, modified Fisher grade, clipping and Ngb levels on days 1, 2, 5, and 7. Sensitivity, specificity, and *Youden* index of day 3 Ngb level for identifying DCI were derived as 73.9%, 72.5%, and 0.46, respectively, based on the best threshold of 8.4 ng/ml. Regardless in good‐grade group or in poor‐grade group, patients having day 3 Ngb level > 8.4 ng/ml has a significantly worse DCI survival rate than those having day 3 Ngb level <=8.4 ng/ml (*p* = .026 and .009, respectively).

**Conclusions:**

Serum Ngb level was significantly elevated in DCI patients. Early stage aSAH Ngb level has the potential of being used as a novel DCI occurrence predictor, especially when Ngb level was combined with WFNS grade.

## Graphical abstract

Although qualitative or semiquantitative clinical grades (such as WFNS grade and modified Fisher grade) are well known to be associated with DCI following aSAH, identification of an easily measurable and more objective biomarker for predicting DCI might be helpful for early prediction and risk mitigation. In the current study, we found that the early stage neuroglobin level was a significant predictor of DCI in aSAH patients. Furthermore, combined use of WFNS grade and Ngb level might be able to better identify aSAH patients with high risk of DCI.

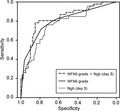

## 1 INTRODUCTION

Aneurysmal subarachnoid hemorrhage (aSAH) is one of the most severe neurosurgical diseases with a high death rate. It generates a large healthcare burden (Etminan et al., 2019; Macdonald & Schweizer, 2017). Delayed cerebral ischemia (DCI) usually occurs at 3–14 days post‐aSAH. It is one of the most common complications of aSAH and is considered a leading cause of death or major disability in SAH (Al‐Mufti et al., 2019; Macdonald, 2014). There is a close connection between the occurrence of DCI and long‐term adverse prognosis in aSAH patients (Duan et al., 2018). Therefore, it is critical to detect early and prevent the occurrence of DCI in the diagnosis and treatment of aneurysms, as it could prevent nonreversible brain damage (Darkwah Oppong et al., 2018; Roh, Morris, & Claassen, 2016). Thus, it could be helpful for the treatment and prevention of poor prognosis of aSAH by identifying the subset of patients with high DCI risk.

The pathogenesis of DCI still remains unclear. In recent research, the role of vasospasm, microvasculature, coagulation, and fibrinolytic systems to DCI are identified (Geraghty & Testai, 2017). Hypoxic‐ischemic brain damage is a critical condition caused by vascular events including cerebral vasospasm, microvessel thrombosis, and microvascular dysfunction and might lead to the development of DCI (Milner et al., 2015; Mutoh et al., 2017; Wagner et al., 2013).

Neuroglobin (Ngb) is a type of hemoprotein specific to the brain, and its production is specifically increased during hypoxic‐ischemic brain damage. Ngb could combine specifically with and reduce excessive NO through allosteric regulation, therefore acting as a neuroprotectant by reducing active oxygen in nervous tissue (Liu et al., 2009; Shang et al., 2006). It has been reported that serum Ngb level increased significantly during brain damage in patients with TBI (Chen et al., 2015) aSAH (Cai et al., 2018), and ischemic stroke (Xue et al., 2017). However, the relationship between Ngb level and occurrence of DCI following aSAH is still unclear. In this study, we sought to investigate whether Ngb level in the early stage of aSAH may help with identifying patients at high risk of DCI.

## 2 MATERIALS AND METHODS

### 2.1 Study population

The authors declare that all data used to support the findings of this study are included within the article. This is a prospective observational cohort study without planned intervention, based on our previous study (Cai et al., 2018) with additionally enrolled patients and analyses of other parameters. Patients admitted to the department of neurosurgery in our hospitals, within 24 hr of SAH onset, and met our inclusion criteria were included in this study. Between March 2017 and September 2017, a total of 126 consecutive patients were enrolled in this study. Furthermore, 42 healthy subjects, at a ratio of 1:3 to the enrolled patients were included.

The inclusion criteria for patients were as follows: (a) patient admitted to hospital within 24 hr of SAH onset; (b) the serum Ngb levels at admission, on the first morning, and on the second morning of hospitalization were obtained; (c) the SAH caused by intracranial aneurysm was confirmed via computed tomography angiography (CTA) or digital subtraction angiography (DSA); (d) clipping or interventional treatment of aneurysm was performed within 48 hr. Exclusion criteria for patients were as follows: (a) the patient was discharged within 30 days and was not able to revisit the hospital for the completion of follow‐up CT scans; (b) the patient had any type of surgery or acute or chronic infection with in the past month; (c) prior onset of SAH or other neurological diseases such as ischemic stroke, hemorrhagic stroke, or severe head trauma; (d) previous use of antiplatelet and anticoagulant drugs, or immunosuppressants; (e) other systematic diseases, such as autoimmune disease, uremia, cirrhosis, cancer, chronic lung diseases, and chronic heart diseases including coronary artery diseases and myocardial infarction (Cai et al., 2018).

The study protocol was designed in accordance with guidelines outlined in the Declaration of Helsinki and approved by the local Institutional Review Boards. Informed consent was obtained from the healthy subjects, the patient or his/her legally authorized representative if the patient cannot give or refuse consent him/herself, in accordance with the Chinese law.

### 2.2 Patient management

American Heart Association and American Stroke Association guidelines were used in this study for clinical management (Connolly et al., 2012) Critical care management followed the guideline from Neurocritical Care Society (Diringer et al., 2011). Hypertensive hypervolemic therapy was performed on patients with symptomatic vasospasm. For poor‐grade patients having severe angiographic vasospasm, target systolic blood pressure was set to 180–220 mmHg (Komotar et al., 2009).

### 2.3 Data collection

Clinical information was collected for each patient. The modified Fisher grade was used to semiquantify the amount of subarachnoid blood presented on the admission CT scan (Claassen et al., 2001). Severity of aSAH was assessed on the basis of the initial Hunt & Hess scale, and World Federation of Neurosurgical Societies (WFNS) grade. Admission WFNS Grade < 3 was considered as good grade and WFNS Grade ≥ 3 was considered as poor grade. The condition of each patient was graded by two neurosurgeons, and a third neurosurgeon will give a final grade if the two surgeons gave very different scores. Serum Ngb level at days 1, 2, 3, 5, and 7 were determined using enzyme‐linked immunosorbent assay (ELISA). The venous blood sample used for determining Ngb level was collected into tubes containing ethylene diamine tetraacetic acid and immediately transferred to the laboratory, where it was centrifuged at 1,000 *g* for 20 min at room temperature. The supernatant was collected and stored in −80°C freezer until analysis. ELISA was performed using a commercial kit (Clone Corp., Houston, TX, USA) according to the manufacturer's instruction. The limit of detection and coefficient of variation were 7.8 pg/ml and 5.8%, respectively.

For the demographically matched healthy volunteers, venous blood was sampled in the morning, at noon, and in the evening of the same day for detecting Ngb level. The average value and standard deviation were obtained for analysis.

For aSAH patients, to detect DCI, brain edema, and intraparenchymal hematoma in a time‐dependant manner, brain CT scans were performed at days 1, 7, 14, 30 postsurgery, in addition to the regular clinically needed CT scans.

The location and size of the aneurysm was determined via CTA or DSA. DCI following aSAH was the primary outcome measured in this study. Patients were followed up until occurrence of DCI, or until one month after aSAH. DCI was diagnosed based on criteria defined in a previous study (Frontera et al., 2009): “(a) clinical deterioration (i.e., a new focal deficit, decrease in level of consciousness, or both), and/or (b) a new infarct on CT that was not visible on the admission or immediate postoperative scan and cannot be attributed to other causes by means of clinical assessment, imaging of the brain, and appropriate laboratory studies.”

### 2.4 Statistical analyses

Statistical analyses were performed with SPSS 17.0 (SPSS Inc). In this study, numerical variables were expressed as mean ± standard deviation and the differences between groups were analyzed by the two‐sample *t* test. Categorical variables were expressed as counts (percentage) and analyzed by either the Pearson *χ* ^2^ test or Fisher exact test. Multivariate logistic regression model was used to evaluate potential predictors of DCI. All available preoperative and operative variables that had univariate association *p* < .10 with the occurrence of DCI were included in the candidate pool of univariate logistic regression analysis. All variables having *p* < .05 from univariate logistic regression analysis were included in multivariate analysis. Then, the backward stepwise multivariate regression was performed to create the final model whereby the least nonsignificant variables were removed from the model one by one, until all remaining variables had *p* < .05. Area under the curve (AUC) model performance was evaluated using the *Z* test. The corresponding sensitivity, specificity, and *Youden* index were also calculated using the best threshold for WFNS grade, modified Fisher grade, and Ngb level, respectively, which were derived from the receiver operating characteristic (ROC) curve analysis. The percentage of patients surviving DCI for 30 days was calculated using Kaplan–Meier method, and the curves of survival rate for patient without DCI were drawn and compared using the log‐rank test. *p* ≤ .05 was considered significant.

## 3 RESULTS

### 3.1 Patient characteristics

A total of 126 patients were included in the analysis. The average age of patients was 53.4 ± 10.7 years old, with 56% being female (71/126). Patient demographics, clinical characteristics, and Ngb levels were compared according to the occurrence of DCI in Table 1. Patients with DCI had significantly higher Ngb levels than those without (*p* < .001) (Table 1). As compared with healthy subjects (*n* = 42, mean Ngb level: 4.7 ± 1.5 ng/ml), the serum Ngb concentrations for aSAH patients in both DCI and non‐DCI groups were significantly higher during the first seven days after aSAH (all *p* < .05).

**Table 1:** Comparison of demographic and clinical data in patients with aSAH according to occurrence of delayed cerebral ischemia

Characteristics	Total (*n* = 126)	DCI group (*n* = 46)	non‐DCI group (*n* = 80)	*p* value
Demographics				
Age, years	53.4 ± 10.7	53.7 ± 9.3	53.3 ± 11.5	.824
Gender, female	71 (56.3%)	24 (52.2%)	47 (58.8%)	.474
Admission clinical finding				
SAP, mmHg	146.1 ± 23.8	149.3 ± 27.3	144.3 ± 21.5	.254
DAP, mmHg	83.8 ± 11.3	84.2 ± 9.9	83.6 ± 12.1	.752
MAP, mmHg	104.6 ± 14.0	105.9 ± 14.3	103.8 ± 13.9	.415
WFNS grade	3 (1–4)	4 (3–5)	1 (1–3)	<.001
Grade I	57 (45.2%)	8 (17.4%)	49 (61.3%)	
Grade II	5 (4.0%）	1 (2.2%)	4 (5.0%)	
Grade III	14 (11.1%）	4 (8.7%)	10 (12.5%)	
Grade IV	29 (23.0%)	15 (32.6%)	14 (17.5%)	
Grade V	21 (16.7%)	18 (39.1%)	3 (3.8%)	
Modified Fisher grade	2 (2–3)	3 (2–4)	2 (2–2)	<.001
Grade I	9 (7.1%)	2 (4.3%)	7 (8.8%)	
Grade II	67 (53.2%)	13 (28.3%)	54 (67.5%)	
Grade III	27 (21.4%)	15 (32.6%)	12 (15.0%)	
Grade IV	23 (18.3%)	16 (34.8%)	7 (8.8%)	
Diabetes mellitus	14 (11.1%)	4 (8.7%)	10 (12.5%)	.513
Smoking history	34 (27.0%)	15 (32.6%)	19 (23.8%)	.281
Aneurysm characteristics				
Aneurysm size, mm	7.4 ± 0.4	7.3 ± 0.5	7.4 ± 0.4	.934
Multiple aneurysms	37 (29.4%)	11 (23.9%)	25 (31.3%)	.629
Anterior location	94 (74.6%)	35 (76.1%)	59 (73.8%)	.772
Surgical treatment				
Clipping	105 (83.3%)	42 (91.3%)	63 (78.8%)	.069
Coiling	21 (16.7%)	4 (8.7%)	17 (21.3%)	
Ngb level, ng/ml				
Day 1	8.2 ± 2.9	9.9 ± 3.7	7.2 ± 1.5	<.001
Day 2	9.6 ± 3.0	11.2 ± 4.0	8.6 ± 1.7	<.001
Day 3	8.4 ± 1.9	9.5 ± 2.2	7.8 ± 1.3	<.001
Day 5	7.6 ± 1.8	8.5 ± 2.1	7.1 ± 1.4	<.001
Day 7	6.8 ± 1.5	7.5 ± 1.8	6.4 ± 1.2	<.001
Mean Ngb^a^	8.1 ± 2.0	9.3 ± 2.5	7.4 ± 1.1	<.001
Interactions				
WFNS grade × Ngb (day 1)	24.2 ± 21.5	40.0 ± 25.2	15.1 ± 11.9	<.001
WFNS grade × Ngb (day 2)	27.7 ± 24.0	44.4 ± 28.2	18.0 ± 14.2	<.001
WFNS grade × Ngb (day 3)	23.7 ± 17.9	36.6 ± 18.7	16.3 ± 12.5	<.001
WFNS grade × Ngb (day 5)	21.7 ± 16.7	33.0 ± 17.4	15.2 ± 12.3	<.001
WFNS grade × Ngb (day 7)	19.2 ± 14.7	29.3 ± 15.6	13.3 ± 10.5	<.001
WFNS grade × mean Ngb	23.3 ± 18.6	36.7 ± 20.5	15.6 ± 12.1	<.001

Abbreviations: DAP, diastolic arterial pressure; MAP, mean arterial pressure; Ngb, neuroglobin; SAP, systolic arterial pressure; WFNS, World Federation of Neurosurgical Societies.

a Mean Ngb value is the average of Ngb day 1, day 2, day 3, day 5, and day 7 Ngb values. Values are *n* (%), mean ± *SD*, and median (25%–75%).

Moreover, half the patients (*n* = 63) with higher mean Ngb (mean value of serum Ngb level on days 1, 2, 3, 5, and 7 was higher than 8.0 ng/ml) had higher WFNS grade, modified Fisher scale, and rate of DCI events than the remaining half patients (*n* = 63) with lower mean Ngb (Table 2).

**Table 2:** Demographics and baseline characteristics of patients classified by the mean Ngb value^a^

Characteristics	High mean Ngb (≥8.0 ng/ml)	Low mean Ngb (<8.0 ng/ml)	*p* value
No. of patients	63	63	
DCI events	39 (61.9%)	7 (11.1%)	<.001
Demographics			
Age, years	54.9 ± 10.5	52.0 ± 10.8	.137
Gender, female	34 (54.0%)	37 (58.7%)	.590
Admission clinical finding			
SAP, mmHg	149.2 ± 23.6	143.1 ± 23.8	.151
DAP, mmHg	85.0 ± 12.0	82.7 ± 10.5	.254
MAP, mmHg	106.4 ± 14.3	102.8 ± 13.6	.154
WFNS grade	4 (3–5)	1 (1–2)	<.001
Grade I	10	47	
Grade II	0	5	
Grade III	12	2	
Grade IV	21	8	
Grade V	20	1	
Modified Fisher grade	3 (2–4)	2 (2–2)	<.001
Grade I	1	8	
Grade II	27	40	
Grade III	13	14	
Grade IV	22	1	
Diabetes mellitus	6 (9.5%)	8 (12.7%)	.571
Smoking history	20 (31.7%)	14 (22.2%)	.229
Aneurysm characteristics			
Aneurysm size, mm	7.3 ± 0.4	7.4 ± 0.5	.837
Multiple aneurysms	17 (27.0%)	20 (31.7%)	.557
Anterior location	50 (79.4%)	44 (69.8%)	.216
Surgical treatment			
Clipping (vs. Coiling)	55 (87.3%)	50 (79.4%)	.232

Abbreviations: DAP, diastolic arterial pressure; MAP, mean arterial pressure; Ngb, neuroglobin; SAP, systolic arterial pressure; WFNS, World Federation of Neurosurgical Societies.

a Based on the mean Ngb value, patients in this study were classified into two groups: those having Ngb ≥ 8.0 ng/ml were included in the high Ngb group, and those having Ngb < 8.0 ng/ml were included in the low Ngb group. Mean Ngb value is the average of Ngb day 1, day 2, day 3, day 5, and day 7 values的平均值. Values are *n* (%), mean ± *SD*, and median (25%–75%).

### 3.2 Ngb levels and DCI

There were 46 patients (36.5%) who experienced DCI within one month after aSAH. Eight variables, such as the WFNS grade, modified Fisher grade, clipping, and Ngb levels on days 1, 2, 3, 5, and 7, had univariate association *p* < .10 with the occurrence of DCI (Table 1), thus used in univariate and multivariate model analyses. Multivariate model analysis revealed that day 3 Ngb level and WFNS grade were both significant predictors associated with the DCI (day 3 Ngb level: odds ratio [OR] = 1.57, 95% confidence interval [CI] = 1.12–2.19, *p* = .009; WFNS grade: OR = 1.81, 95%CI = 1.33–2.47, *p* < .001) (Table 3). Day 3 Ngb level and WFNS grade remained significant factors after adjusting for the interaction factor [WFNS grade × Ngb (day 3)] (Table 3).

**Table 3:** Multivariate model analysis of delayed cerebral ischemia with predictors

Predictor^a^	Univariate analysis				Multivariate analysis^b^	
	DCI (*n* = 46)	non‐DCI (*n* = 80)	OR (95% CI)	*p* value	OR (95% CI)	*p* value
Components of Model 1						
WFNS grade	4 (3–5)	1 (1–3)	2.19 (1.64–2.91)	<.001	1.81 (1.33–2.47)	<.001
Modified Fisher grade	3 (2–4)	2 (2–2)	2.89 (1.78–4.68)	<.001	–	–
Clipping	42 (91.3%)	63 (78.8%)	2.83 (0.89–9.01)	.078	–	–
Ngb (day 1), ng/ml	9.9 ± 3.7	7.2 ± 1.5	1.56 (1.26–1.93)	<.001	–	–
Ngb (day 2), ng/ml	11.2 ± 4.0	8.6 ± 1.7	1.47 (1.20–1.80)	<.001	–	–
Ngb (day 3), ng/ml	9.5 ± 2.2	7.8 ± 1.3	1.94 (1.44–2.62)	<.001	1.57 (1.12–2.19)	.009
Ngb (day 5), ng/ml	8.5 ± 2.1	7.1 ± 1.4	1.57 (1.24–2.00)	<.001	–	–
Ngb (day 7), ng/ml	7.5 ± 1.8	6.4 ± 1.2	1.72 (1.30–2.33)	<.001	–	–
mean Ngb, ng/ml	9.3 ± 2.5	7.4 ± 1.1	2.19 (1.49–3.21)	<.001	–	–
Components of Model 2						
WFNS grade	4 (3–5)	1 (1–3)	2.19 (1.64–2.91)	<.001	1.81 (1.33–2.47)	<.001
Ngb (day 3), ng/ml	9.5 ± 2.2	7.8 ± 1.3	1.94 (1.44–2.62)	<.001	1.57 (1.12–2.19)	.009
WFNS grade × Ngb (day 1)	40.0 ± 25.2	15.1 ± 11.9	1.08 (1.05–1.11)	<.001	–	–
WFNS grade × Ngb (day 2)	44.4 ± 28.2	18.0 ± 14.2	1.07 (1.04–1.10)	<.001	–	–
WFNS grade × Ngb (day 3)	36.6 ± 18.7	16.3 ± 12.5	1.09 (1.05–1.12)	<.001	–	–
WFNS grade × Ngb (day 5)	33.0 ± 17.4	15.2 ± 12.3	1.08 (1.05–1.11)	<.001	–	–
WFNS grade × Ngb (day 7)	29.3 ± 15.6	13.3 ± 10.5	1.09 (1.06–1.13)	<.001	–	–
WFNS grade × mean Ngb	36.7 ± 20.5	15.6 ± 12.1	1.08 (1.05–1.11)	<.001	–	–

Abbreviations: DCI, delayed cerebral ischemia; Ngb, neuroglobin; OR, odds ratio; WFNS, World Federation of Neurosurgical Societies.

a Predictors of Model 1 included the preoperative and operative items listed in Table 1 that had *p* < .10 (except the interaction variables “WFNS grade × Ngb”). Predictors of Model 2 included the variables which were significant in the multivariate model analysis of Model 1, and the interaction variables.

b Multivariate analysis: all variables having *p* < .05 from univariate analyses were included in multivariate analysis. The backward stepwise multivariate regression was performed to create the final model whereby the least nonsignificant variable was removed from the model one by one, until all remaining variables had *p* < .05.

**Figure 1:**
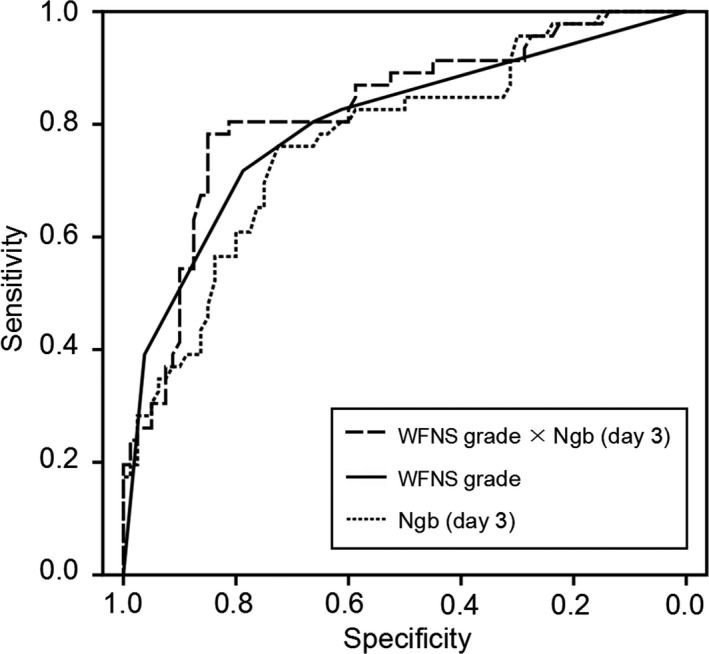
ROC curve analysis comparing WFNS grade, day 3 Ngb level and interaction factor “WFNS grade × Ngb (day 3)” on predicting DCI in SAH patients. The AUCs of WFNS grade and day 3 Ngb level were 0.80 (95%CI 0.72–0.88) and 0.77 (95%CI 0.69–0.86), respectively, and were comparable (*Z* = 0.439, *p* = .661). The AUC of Ngb (day 3) was significantly increased after the WFNS grade was combined with it [interaction: WFNS grade × Ngb (day 3)], although the difference was not statistically significant [0.77(95%CI = 0.69–0.86) vs. 0.82(95%CI = 0.74–0.90), *Z* = 2.552, *p* = .391]. The sensitivity, specificity, and *Youden* index of day 3 Ngb level for identifying DCI were derived as 73.9%, 72.5%, and 0.46, respectively, based on the best threshold of 8.4 ng/ml. The factor WFNS grade × Ngb (day 3) might have a better predictive performance of DCI (sensitivity = 78.3%, specificity = 85.0%, and best threshold = 32.2) than that of Ngb (day 3)

Receiver operating characteristic curve analysis also confirmed that WFNS grade (AUC = 0.80, 95%CI = 0.72–0.88) and day 3 Ngb level were both strong predictors of DCI. The predictive performance of day 3 Ngb level for the occurrence of DCI was represented as AUC = 0.77 (95%CI = 0.69–0.86), and the sensitivity, specificity, and *Youden* index were derived as 73.9%, 72.5%, and 0.46, respectively, based on the best threshold of 8.4 ng/ml. The predictive power of WFNS grade and day 3 Ngb level were comparable (*Z* = 0.439, *p* = .661) (Figure 1).

Combined use of WFNS grade and Ngb level might be able to better identify aSAH patients at high risk of DCI. Comparisons of the interaction factors based on WFNS garde and Ngb between the patients of DCI group and non‐DCI group were showed in Table 1. The AUC of Ngb was increased after the WFNS grade was combined with it [interaction: WFNS grade × Ngb (day 3)], although the difference was not statistically significant [0.77(95%CI = 0.69–0.86) vs. 0.82(95%CI = 0.74–0.90), *Z* = 2.552, *p* = .391] (Figure 1). The factor WFNS grade × Ngb (day 3) might have a better predictive performance of DCI (sensitivity = 78.3%, specificity = 85.0%, and best threshold = 32.2) than Ngb (day 3).

### 3.3 DCI survival analysis of patients with and without elevated day 3 Ngb level

**Figure 2:**
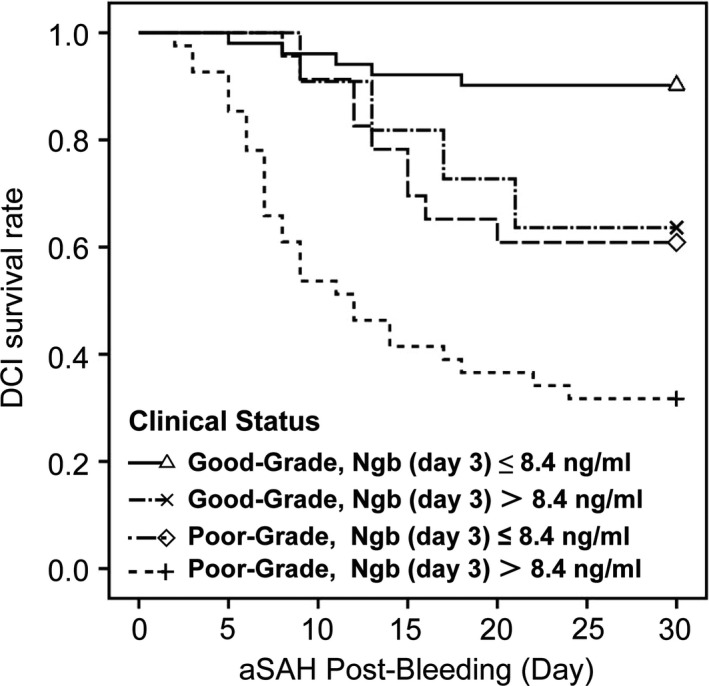
The Kaplan–Meier curve of the 30‐day DCI survival rate of patients. The Kaplan–Meier curve shows a 30‐day DCI survival rate (i.e., the rate of patients without DCI) of 90.2%(46/51) for good‐grade patients having day 3 Ngb level ≤ 8.4 ng/ml, of 63.6%(7/11) for good‐grade patients having day 3 Ngb level > 8.4 ng/ml, of 60.9%(14/23) for poor‐grade patients having day 3 Ngb level ≤ 8.4 ng/ml, and of 31.7%(13/41) for poor‐grade patients having day 3 Ngb level > 8.4 ng/ml. The log‐rank test showed *p* < .001. Post hoc log‐rank testing revealed that regardless in good‐grade group or in poor‐grade group, patients having day 3 Ngb level > 8.4 ng/ml has a significantly worse DCI survival rate than those having day 3 Ngb level <=8.4 ng/ml (*p* = .026 and 0.009, respectively

Among good‐grade patients (WFNS Grade < 3, *n* = 62), 11 (17.7%) had day 3 Ngb level > 8.4 ng/ml. Among poor‐grade patients (WFNS Grade ≥ 3, *n* = 64), 41 (64.1%) had day 3 Ngb level > 8.4 ng/ml. The Kaplan–Meier curve shows a 30‐day DCI survival rate (i.e., the rate of patients without DCI) of 90.2%(46/51) for good‐grade patients having day 3 Ngb level ≤ 8.4 ng/ml, of 63.6%(7/11) for good‐grade patients having day 3 Ngb level > 8.4 ng/ml, of 60.9%(14/23) for poor‐grade patients having day 3 Ngb level ≤ 8.4 ng/ml, and of 31.7%(13/41) for poor‐grade patients having day 3 Ngb level > 8.4 ng/ml. Regardless in good‐grade group or in poor‐grade group, patients having day 3 Ngb level > 8.4 ng/ml has a significantly worse DCI survival rate than those having day 3 Ngb level ≤ 8.4 ng/ml (*p* = .026 and .009, respectively). Moreover, the DCI survival rate for good‐grade patients having Ngb (day 3) >8.4 ng/ml was not significantly better than that of poor‐grade patients having Ngb (day 3) ≤8.4 ng/ml (*p* = .796) (Figure 2).

## 4 DISCUSSION

Delayed cerebral ischemia usually occurs within two weeks of aSAH and is a common and severe complication of aSAH. The results of medicinal treatment for DCI are often not satisfactory, and it usually leads to permanent damage to neurological functions, resulting in disability or even death of SAH patients (Al‐Mufti et al., 2019; Ayling, Ibrahim, Alotaibi, Gooderham, & Macdonald, 2016; Budohoski et al., 2012; Macdonald, 2014; Ostergaard et al., 2013). If early prognosis of the risk of DCI could be achieved, optimized care and improved allocation of healthcare resources might be facilitated. Although qualitative or semiquantitative clinical grades (such as WFNS grade and modified Fisher grade) are well known to be associated with DCI following aSAH, identification of an easily measurable and more objective biomarker for predicting DCI might be helpful for early prediction and risk mitigation. Ngb, as a nervous system‐specific hemoprotein, is easy to obtain and to be integrated into daily clinical practice; it also has applicable sensitivity and specificity which were comparable with WFNS grade. As Ngb is more quantitative and objective than clinical grades, it may be able to reflect small changes in the physical condition of a patient that could not be reflected in clinical grades, which could lead to changes in treatment plan. In the current study, we found that the day 3 Ngb level was a significant predictor of DCI in aSAH patients.

The mechanism of DCI is still unclear. One critical process that could lead to DCI is hypoxic‐ischemic brain damage, which might be caused by vascular events including cerebral vasospasm, microvessel thrombosis, and microvascular dysfunction (Milner et al., 2015; Mutoh et al., 2017; Wagner et al., 2013). Biomarkers related to inflammatory reaction (Chamling et al., 2017), microthrombosis (Boluijt, Meijers, Rinkel, & Vergouwen, 2015), cerebral metabolism (Rostami et al., 2018), and brain injury might be used to predict occurrence of DCI in aSAH patients (Jung, Lange, Zimmermann, & Seifert, 2013). However, technologies such as detection of related biomarkers may eventually be used in goal‐oriented therapy, with the aim of creating an optimal physiological environment for the comatose injured brain. There is still a need for more reliable biomarkers and better use of the already established ones for a more effectively guided clinical diagnosis and assessment of aSA (Carpenter et al., 2015). As a intracranial‐specific hemoprotein, Ngb has very high oxygen affinity and is highly expressed during hypoxic‐ischemic brain damage. It plays an important role in neurological protection during hypoxic‐ischemic brain damage (Liu et al., 2009; Shang et al., 2006). After traumatic brain injury, Ngb might play the role of oxygen sensor and function to modulate signal pathway, weaken oxidative stress reaction, and maintain normal functions of mitochondria. In hypoxic‐ischemic brain injury, often a lot of toxic compounds, active free oxygen, and nitrogen radicals accumulate in the brain and Ngb can clean up these free radicals. Using a rat focal brain injury model, Shang et al. (2006) found increased Ngb level at both 12 and 36 hr postinjury. Furthermore, by over expressing Ngb with recombinant adenovirus, both the volume of necrotic tissue and number of apoptotic cells decreased, as conditioned over expression of Ngb can significantly improve the survival rate of neurons posttraumatic brain injury. Based on animal and human studies, the Ngb level is positively related to the degree of brain injury in TBI, aSAH, and ischemic stroke (Cai et al., 2018; Chen et al., 2015; Shang et al., 2006).

In the current study, the incidence rate of DCI was 36.5% for all patients. The DCI incidence rate was 14.0%(8/57), 20.0%(1/5), 28.6%(4/14), 48.3%(14/29), and 85.7%(18/21) for WFNS Grade I‐V patients, respectively. Published articles reported that the incidence rate of DCI ranged from 30% to 40% in all grade patients and 0%–22% in good‐grade patients (Budohoski et al., 2014; Zijlmans et al., 2018). The DCI incidence rate reported in this study matched with that reported previously. Our study also finds that the mean Ngb level elevated on day 1, peaked on day 2, and then decreased gradually during the first 7 days in aSAH patients. Serum Ngb level in the early stage of aSAH was significantly higher in patients who experienced DCI than those who did not. Multivariate logistic regression analysis showed that day 3 Ngb level might be a more significant predictor than Ngb levels on days 1, 2, 5, and 7. The predicting performance of day 3 Ngb level on DCI was comparable to that of WFNS grade. Our analysis showed an applicable sensitivity (73.9%) and specificity (72.5%) for aSAH patients with day 3 Ngb level > 8.4 ng/ml and had DCI within one month after aSAH, indicating they might need to be more closely monitored. The AUC of Ngb (day 3) was significantly increased after the WFNS grade was combined with it [interaction: WFNS grade × Ngb (day 3)], although the difference was not statistically significant (*Z* = 2.552, *p* = .391). The factor WFNS grade × Ngb (day 3) might have a better predictive performance of DCI (sensitivity = 78.3%, specificity = 85.0%, and best threshold = 32.2) than Ngb (day 3). Combined use of WFNS grade and Ngb level might be able to better identify aSAH patients with high risk of DCI. Moreover, both in good‐grade and poor‐grade groups, patients having day 3 Ngb level > 8.4 ng/ml had significantly worse DCI survival rate than those having day 3 Ngb level ≤ 8.4 ng/ml. Our data suggest that the predictive value of Ngb for DCI might not be only limited to poor‐grade patients, but also applicable to good‐grade patients.

Our study has a number of limitations. In addition to its observational design, one limitation is that it takes 3–4 hr to obtain the result of Ngb test, while the head CT images are available to be viewed within 30 min and the WFNS grade can be immediately obtained from physical examination. Therefore, it is necessary to reduce the time by developing a faster and standardized Ng measurement method. Another limitation is that serum Ngb levels but not cerebrospinal fluid (CSF) Ngb levels were measured. Previous research reported that after brain injury, both serum and CSF Ngb levels increased rapidly and they are significantly related (Liu et al., 2009). It has also been reported that after whole‐brain perfusion damage, serum and cortical Ngb levels are significantly related, and serum Ngb level can be used to monitor brain damage in ischemic brain diseases (Shang et al., 2006). Future studies to determine Ngb levels in CSF may be needed to verify the results of the current study.

## 5 CONCLUSIONS

Ngb level in early stage of aSAH may be used as an easily obtained biomarker for predicting the occurrence DCI. Serum Ngb level was significantly higher in DCI patients than that of non‐DCI patients. The day 3 Ngb level may have a predictive performance of DCI (sensitivity = 73.9%, specificity = 72.5%, and best threshold = 8.4 ng/ml) comparable with WFNS grade. Patients with day 3 Ngb level > 8.4 ng/ml had significantly worse DCI incidence rate than those with day 3 Ngb level ≤ 8.4 ng/ml regardless if they have good or poor WFNS grade.

## CONFLICT OF INTEREST

None declared.

## Data Availability

The data that support the findings of this study are available from the corresponding author upon reasonable request.
